# Supplementary material for: Mott–Hubbard insulating state for the layered van der Waals FePX3 (X: S, Se) as revealed by NEXAFS and resonant photoelectron spectroscopy
Source: Sci Rep. 2022 Jan 14;12:735. doi: 10.1038/s41598-021-04557-1 (PMC8760309; doi:10.1038/s41598-021-04557-1)
Supplement: Supplementary file 1 — Supplementary Information. [file 41598_2021_4557_MOESM1_ESM.pdf]

Supplementary information for the manuscript:

"Mott-Hubbard insulating state for the layered van der Waals $FePX_3$ (X: S, Se) as revealed by NEXAFS and resonant photoelectron spectroscopy"

Yichen Jin[1], Mouhui Yan[1], Tomislav Kremer[2], Elena Voloshina[1,2,3,*], and Yuriy Dedkov[1,2,3,]**

[1]Department of Physics, Shanghai University, 99 Shangda Road, 200444 Shanghai, P. R. China
[2]Institut für Chemie und Biochemie, Freie Universität Berlin, 14195 Berlin, Germany
[3]Centre of Excellence ENSEMBLE3 Sp. z o. o., Wolczynska Str. 133, 01-919 Warsaw, Poland

[*]elena.voloshina@icloud.com
[**]yuriy.dedkov@icloud.com

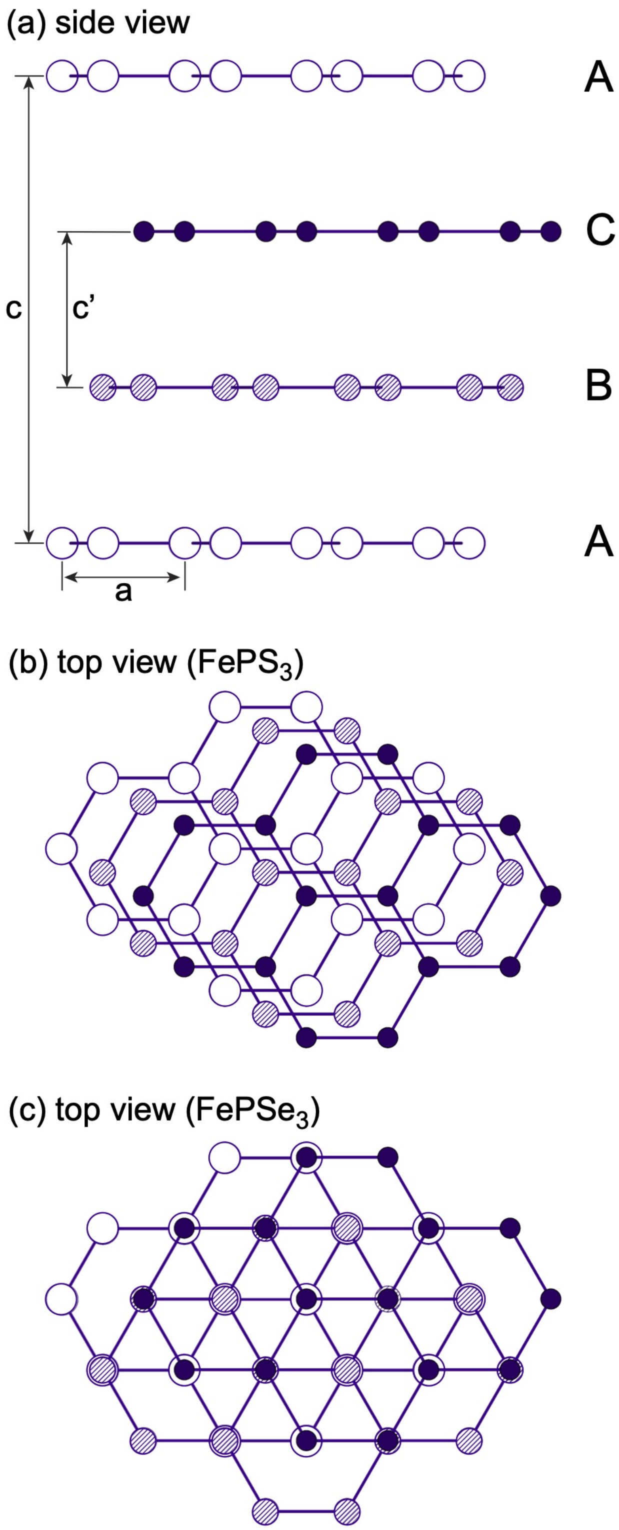

Figure S1. *ABC*-sequence of layers in 3D $FePX_3$. For simplicity, only Fe-ions are shown. Atoms of different layers are shown with spheres of different size and style. In (a) the in-plane and out-of-plane lattice constants are indicated with letters a and c, respectively; $c'=c/3$ is the distance between single layers; (b) and (c) highlight the difference between stackings in $FePS_3$ and $FePSe_3$. Images are created using CrystalMaker X (Version: 10.7.0), http://www.crystalmaker.com.

Table S1. Results for the 3D bulk structure of $FePX_3$ obtained for different magnetic states: E (in eV) is the total energy; ΔE (in meV) is the energy difference between the energy calculated for the different magnetic states and the energy calculated for the lowest energy structure; a, c (in Å) are the in-plane and out-of-plane lattice constants; $c' = c/3$ (in Å) is the distance between single layers (cf. Figure S1).

System	Magn. state	E	ΔE	a	c	c'
$FePS_3$	AFM	−160.799	0	5.936	19.611	6.537
	FM	−160.646	153	5.948	19.650	6.550
	NM	−154.315	6485	5.720	18.808	6.269
$FePSe_3$	AFM	−147.449	0	6.279	19.863	6.621
	FM	−147.357	92	6.279	19.865	6.622
	NM	−141.099	6350	6.071	19.260	6.420

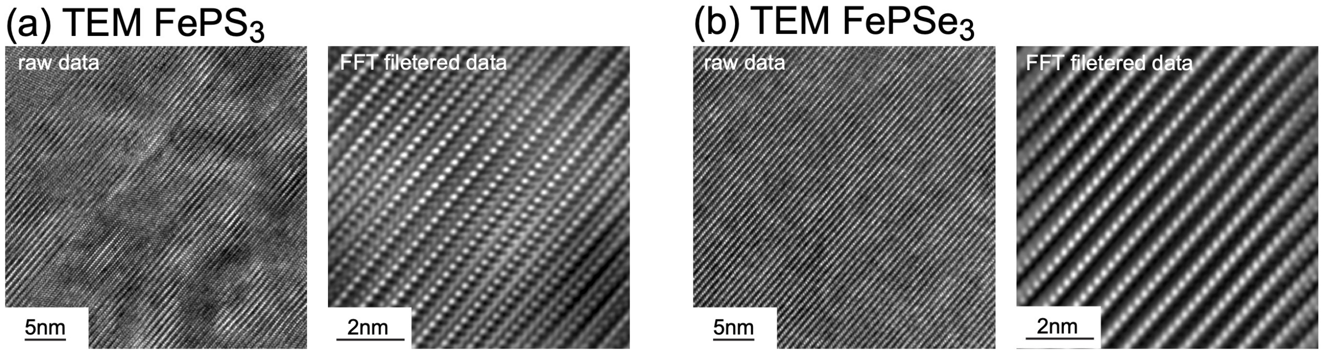

Figure S2. High-resolution TEM images of bulk (a) $FePS_3$ and (b) $FePSe_3$. Images are compiled using Adobe Illustrator 2022 (Version 26.0.2), https://www.adobe.com.

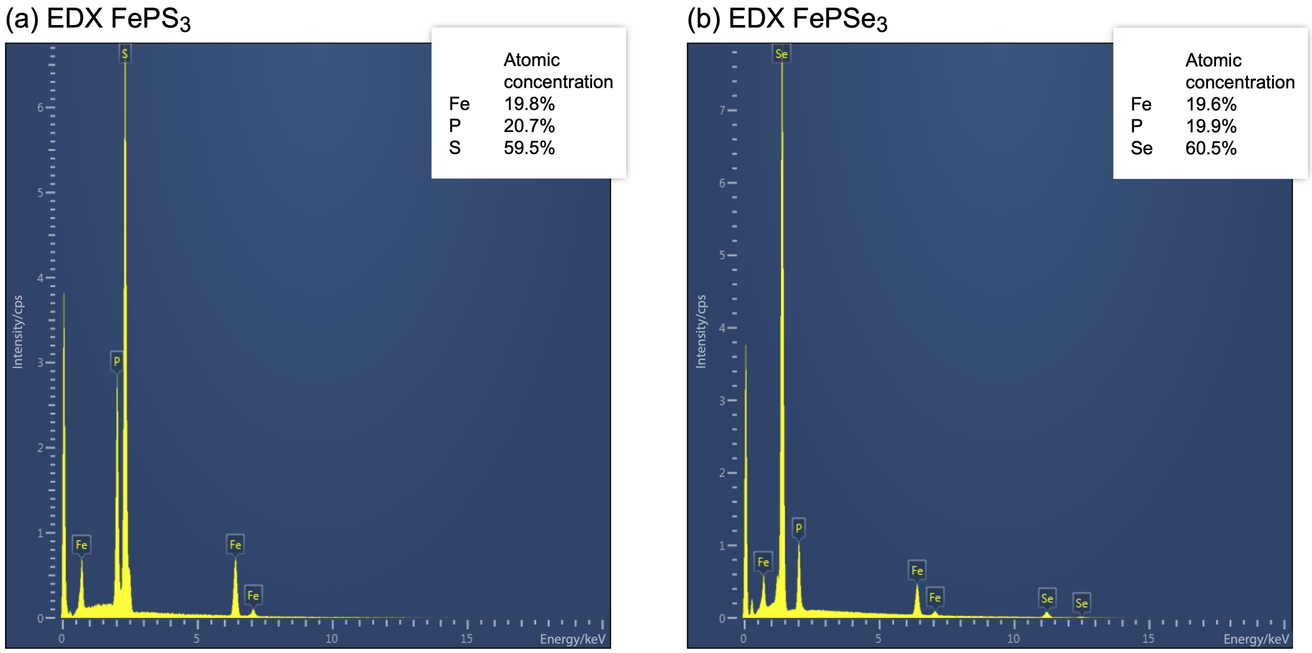

Figure S3. Results of the EDX analysis for (a) $FePS_3$ and (b) $FePSe_3$. Images are created using Igor Pro (Version 9), https://www.wavemetrics.com.

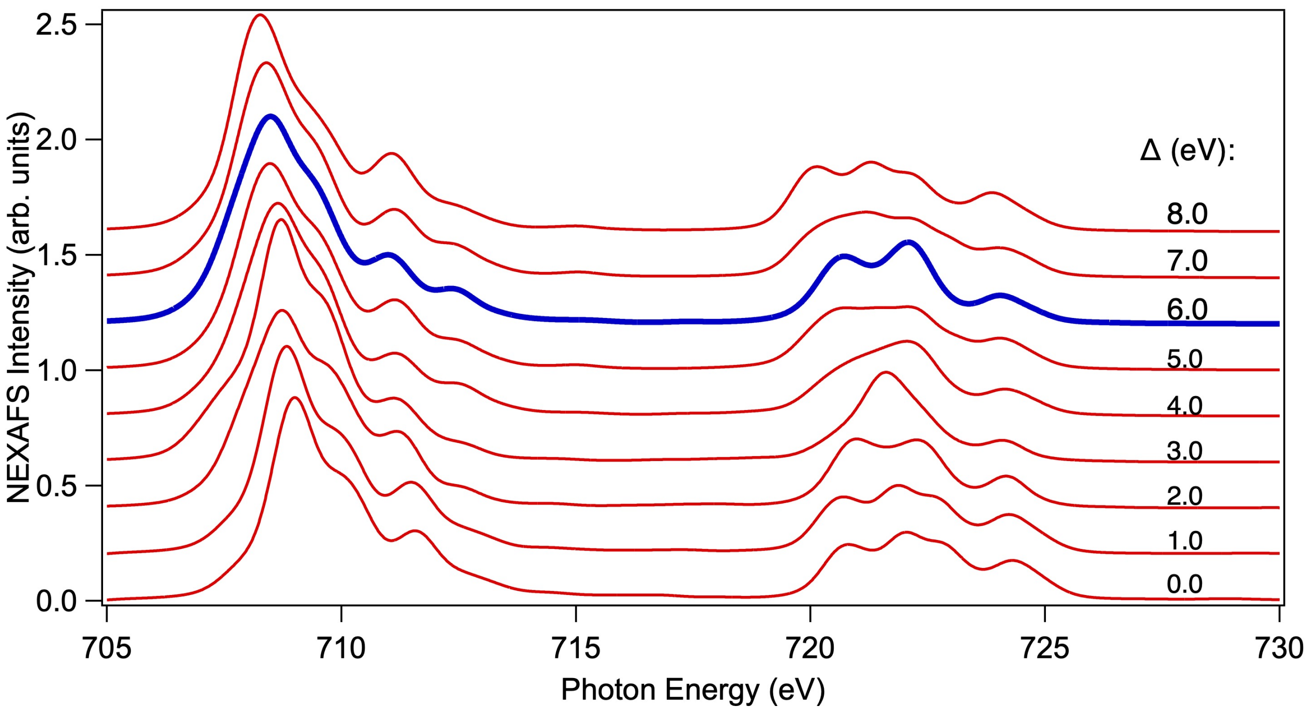

Figure S4. Simulated Fe $L_{2,3}$ NEXAFS spectra of $FePX_3$ with $10Dq$=0.3 eV, U_{dd}=3 eV and different values of Δ marked for every spectra. Images are created using Igor Pro (Version 9), https://www.wavemetrics.com.
